# Bilateral Wilms’ Tumor With Different Responses to Preoperative Chemotherapy

**DOI:** 10.7759/cureus.30593

**Published:** 2022-10-22

**Authors:** Wadah Qari, Alyazid Alzahrani, Mohammed Alzahrani, Yasir Saleh, Abdulrahim Almasabi, Osama Bawazir

**Affiliations:** 1 General Surgery, Umm Al-Qura University, Makkah, SAU; 2 Medicine, Umm Al-Qura University, Makkah, SAU; 3 Surgery, Umm Al-Qura University, Makkah, SAU; 4 Pediatric Surgery and Pediatric Urology, King Faisal Specialist Hospital & Research Centre, Makkah, SAU

**Keywords:** nephron-sparing surgery, case report, small blue cell tumor, frozen section, nuclear scan, wilms’ tumor

## Abstract

Wilms’ tumor (WT) is a common type of primary renal tumor in the pediatric population that can equally affect both sides. Herein, we describe a case of bilateral Wilms’ tumor (BWT) in a three-year-old male with different responses to preoperative chemotherapy in the two kidneys. The left kidney mass responded poorly to chemotherapy than the right kidney, which raised the suspicion of coexisting benign disease or congenital anomaly. However, it was ruled out by preoperative nuclear scan and ultrasound-guided frozen section biopsy taken from the left kidney mass. The report of a frozen section on hematoxylin and eosin (H&E) stain was positive for the small blue cell tumor. The patient was managed successfully with a total nephrectomy of the right kidney and nephron-sparing surgery (NSS) on the left kidney. The postoperative period was uneventful and was managed successfully with radiotherapy. Despite many challenges faced in the management of bilateral Wilms’ tumor, surgery is the most preferable mode of therapy with chemotherapy and radiotherapy being effective in certain cases. The patient was followed up till no signs of recurrence or metastasis were observed.

## Introduction

Wilms’ tumor (WT) is a common renal cancer in the pediatric age group that affects one in every 10,000 children [1]. It was first described and documented by the German physician Dr. Max Wilms in the year 1899 [2]. Bilateral tumors are detected in about 5%-10% of cases of Wilms’ tumor [1]. Bilateral Wilms’ tumor (BWT) can either occur synchronously or metachronously [1]. The current treatment protocol for BWT recommends neoadjuvant chemotherapy (NACT), followed by tumor assessment at six weeks. Thereafter, NACT is continued for another six weeks for maximum shrinkage of the tumor, after which nephron-sparing surgery (NSS) is scheduled [3-5]. Herein, we present a case of BWT in a three-year-old male with different histopathological findings.

## Case presentation

A three-year-old male with no significant clinical history presented with a complaint of painful abdominal lumps observed after an incidence of falling. Syndromic features such as aniridia and gigantism can be mentioned as examined/absent. Abdominal ultrasonography (USG) showed the presence of bilateral renal masses. There was no reported history of urinary tract infections and no family history of cancer and consanguinity.

Routine blood investigations were within normal limits. Abdominal USG revealed a large tissue mass arising from the right kidney measuring 15.9 × 9.4 × 9.6 cm with adjacent septated fluid (likely to be hematoma). The inferior vena cava (IVC) was patent. The left tumor was observed using computed tomography (CT). Its dimensions were 0.9 × 1.6 × 1.4 cm.

**Figure 1:**
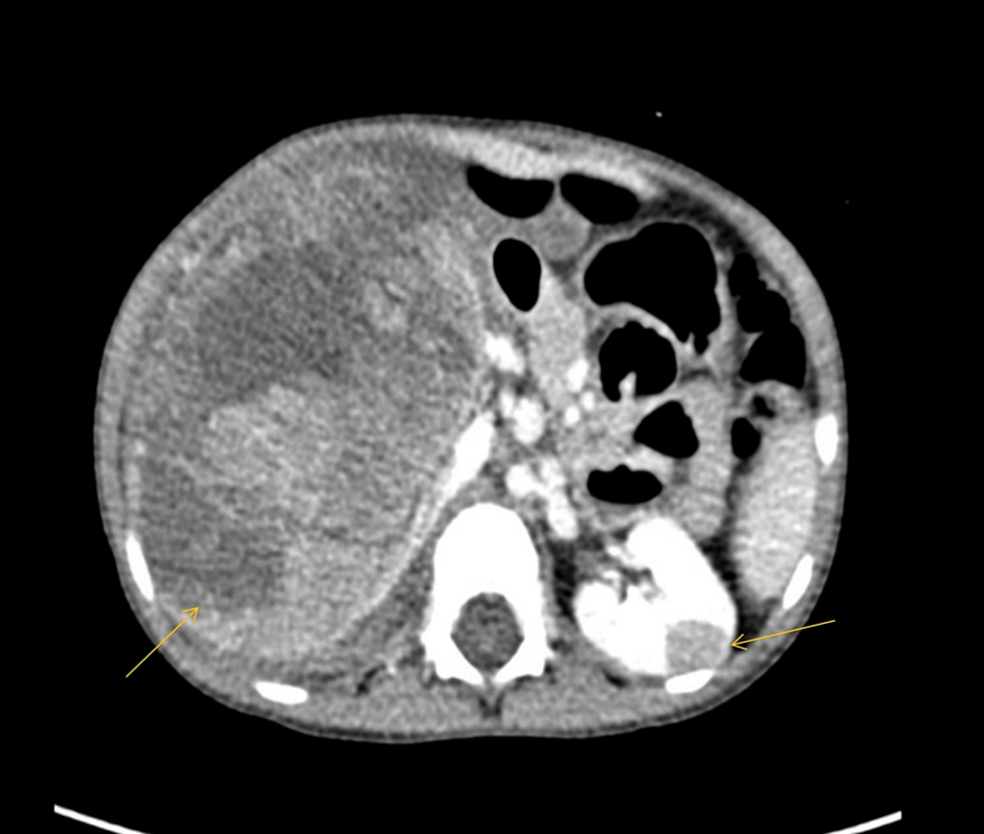
Huge renal mass in the right kidney and small nodules in the mid-left kidney (arrows).

Thereafter, abdominal CT was performed, which showed BWT (stage V) with evidence of tumor rupture on the right side. A small mass with normal-looking parenchyma was observed on the left kidney. Other metastatic workups were negative (Figure 1).

USG-guided biopsy samples were taken from both tumors. Both samples confirmed the histology of Wilms’ tumor. Following confirmation, NACT was started for six weeks with vincristine, dactinomycin, and doxorubicin.

**Figure 2:**
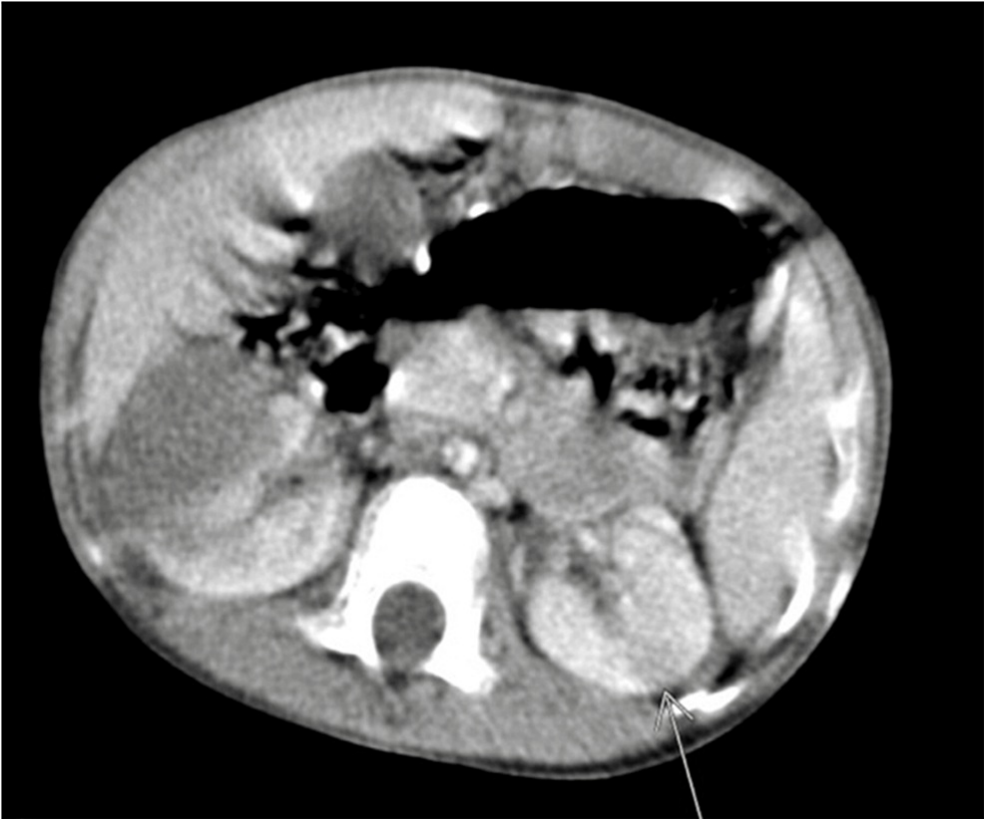
CT scan after six cycles of chemotherapy showed a marked reduction in the right renal tumor and minimal changes in the left tumor (arrow indicates mass in the left kidney). CT: computed tomography

Status post-chemotherapy for six weeks with a repeat CT showed marked interval reduction of the right kidney mass from 11.7 × 8.0 × 11.8 cm to 4.6 × 2.9 × 6.9 cm with no significant change in the left-sided mid-pole lesion from 0.9 × 1.6 × 1.4 cm to 0.9 × 1.5 × 1.2 cm (Figure 2).

**Figure 3:**
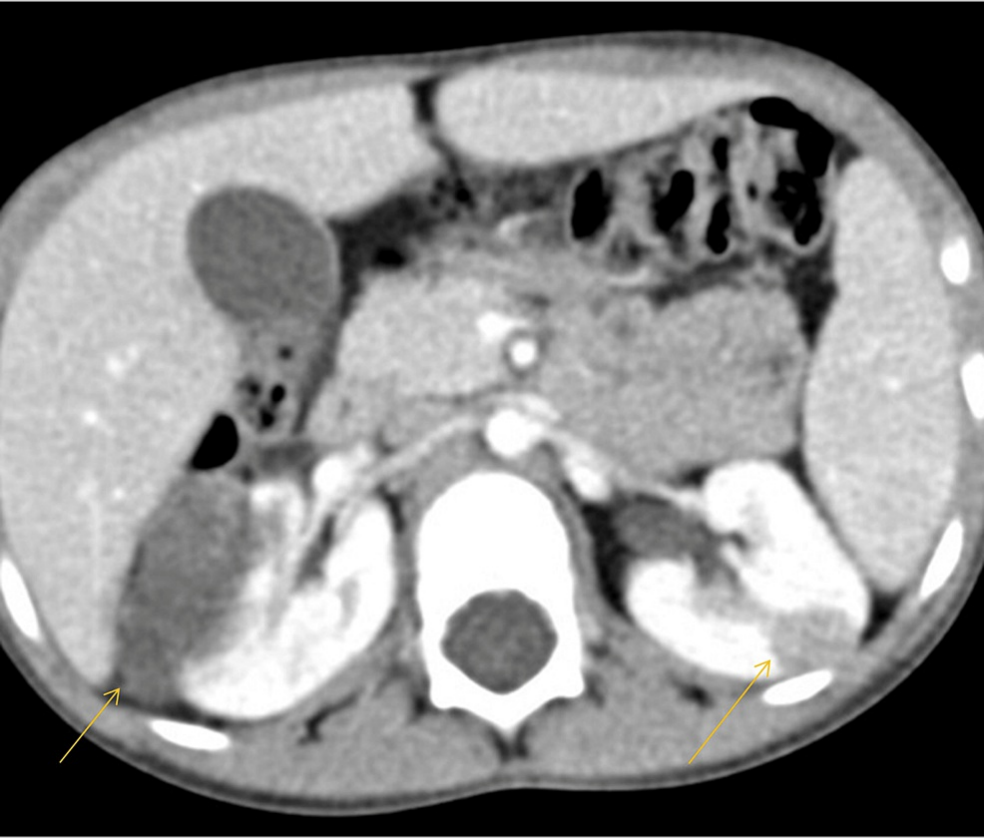
After 12 weeks of chemotherapy, the left renal mass did not change, while a marked reduction was observed in the right renal mass (arrows).

After reviewing the case, a multidisciplinary team decided to continue chemotherapy for another six weeks. Following a total of 12 weeks of chemotherapy, the abdominal CT scan revealed poor chemotherapy response of the left kidney lesion unlike the right kidney mass (Figure 3). Magnetic resonance imaging (MRI) revealed a right renal mass suggestive of Wilms’ tumor; however, for the left kidney mass, the differential diagnosis included splenorenal fusion anomalies, lymphoma, or cyst. A nuclear scan excluded the diagnosis of splenorenal fusion anomaly.

The team further decided to proceed with surgery with a plan of performing NSS. Prior to surgery, a USG-guided biopsy was taken from the left renal mass by an interventional radiologist and sent for the frozen section to rule out malignancy.

A wide right transverse supraumbilical incision was made through a transperitoneal approach. The liver and peritoneum were palpated. Following kocherization of the duodenum, the colon was mobilized from the white line of Toldt, and the right kidney was exposed and inspected. The renal vessels, ureter, and IVC were isolated, with each on a vessel loop.

A complete dissection of the right kidney was done. The result of the left kidney biopsy showed round blue cells. Thus, the abdominal incision was also extended to the left side, and the left kidney was exposed. The same procedure was followed on the right side.

**Figure 4:**
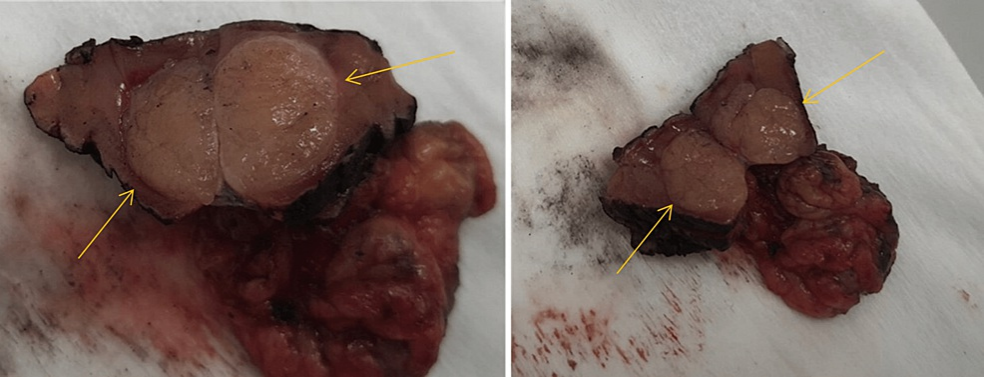
Specimen of the partial left nephrectomy showing the mass with adequate margin grossly (arrows).

After the complete dissection of the left kidney, a bulldog clamp was applied over the left renal vessels, and wedge resection of the left kidney tumor using diathermy and argon beam laser was done. The mass was then sent for a frozen section biopsy (Figure 4), which came with negative margins.

Unfortunately, the remaining 20% in the posterior and inferior part of the right kidney was healthy, and a total nephroureterectomy (NU) was performed with partial adrenalectomy, followed by retrocaval lymph node (LN) biopsy. Primary closure was done over oxidized cellulose impacted on the tumor bed on the left side. Warm ischemia time was nine minutes. Hemostasis was secured, and a left flank drain was inserted. Closure of the abdomen was done in layers, and the dressing was applied. The patient was shifted to the intensive care unit.

Postoperative course

Recovery was uneventful, with normal renal function throughout the postoperative period, and the patient was discharged on the eighth day. Follow-up for 10 months (once every two months) in the clinic showed persistent improvement with no recurrence of the tumor as observed in ultrasound images.

Histopathology results showed the presence of BWT with favorable histology, intermediate risk, negative margin, and negative lymph node. The patient started to receive postoperative radiotherapy after two weeks of surgery.

## Discussion

As per the recommendations of the Children’s Oncology Group (COG), patients with BWT should start treatment with NACT, followed by repeat imaging at an interval of six weeks [6]. If chemotherapy reduces the tumor by 50% or more, a bilateral open biopsy should be performed, followed by additional chemotherapy for a maximum period of another 12 weeks [6,7]. Thereafter, NSS should be planned. The aim of NSS is to remove the renal tumor completely with the preservation of functional renal tissue as much as possible [6,7]. Table 1 enlists similar cases of BWT reported in the literature, along with their case findings, adopted treatment, and final outcomes.

**Table 1:** List of similar cases of bilateral Wilms’ tumor reported in the literature. WT: Wilms’ tumor; DD-4A: vincristine, doxorubicin, and dactinomycin; NSS: nephron-sparing surgery; RAL: robotic-assisted laparoscopy; RN: radical nephrectomy; PN: partial nephrectomy; SOL: space-occupying lesion; GFR: glomerular filtration rate; NACT: neoadjuvant chemotherapy; ActD: actinomycin D; VCR: vincristine; DOX: doxorubicin; CECT: contrast-enhanced computed tomography; DMSA: dimercaptosuccinic acid; BWT: bilateral Wilms’ tumor; NWTS: National Wilms’ Tumor Study; CT: computed tomography; SIOP: International Society of Paediatric Oncology; RCC: renal cell carcinoma; TNM: tumor, node, metastasis

Case findings	Treatment	Final outcomes	Source
A non-syndromic 12-month-old male reported bilateral multifocal WT.	Twelve weeks of preoperative chemotherapy (regimen DD-4A, which includes vincristine, doxorubicin, and dactinomycin) to shrink the tumors at maximum; then, bilateral NSS was performed with successful staging.	Preoperative chemotherapy reduced WT by at least 50%-60%. Bilateral NSS successfully resected the tumors, preserving renal function.	Bowen et al., (2018) [4]
A three-year-old male presented with bilateral renal WT.	Preoperative chemotherapy (according to protocol SIOP-UMBRELLA-2016). RAL approach for left RN and right PN was performed. Also, left flank radiotherapy and chemotherapy for 27 weeks postoperation.	No intraoperative or postoperative complications (right kidney stage: stage III; left kidney stage: stage I).	Sala et al., (2020) [5]
A seven-month-old male was reported with a SOL on both the right and left kidney, suggestive of WT with rhabdomyomatous differentiation. GFR was 54 mL/minute for the left kidney and 46 mL/minute for the right kidney.	Five cycles of NACT (ActD, VCR, and DOX). Open left PN was performed. Open right PN was done after one month. Adjuvant chemotherapy of 24 cycles continued with regular follow-up for 24 months with no recurrence to date.	Post-chemotherapy, CECT showed partial response. DMSA scan after bilateral PN resulted in 40% and 60% split functions for the right and left kidneys, respectively. Postoperative GFR was 45 mL/minute for the left kidney and 31 mL/minute for the right kidney.	Arora et al., (2016) [6]
A 13-month-old male reported SOL in both kidneys and was confirmed for BWT. GFR was 45 mL/minute for the left kidney and 23 mL/minute for the right kidney.	Four cycles of NACT as per the NWTS protocol, with two additional cycles given. A left PN was performed. A right PN was done one month later. Twenty-four cycles of adjuvant chemotherapy in addition to adjuvant radiotherapy were given.	Post-chemotherapy CT scan showed partial response. The complication that occurred was chemotherapy intolerance with severe neutropenia managed conservatively. Postoperative GFR was 35 mL/minute for the left kidney, whereas the right kidney was nonfunctioning.	Arora et al., (2016) [6]
A two-year-old male was found to have bilateral renal masses.	Preoperative chemotherapy (SIOP-2001 guidelines) with VCR injection 0.75 mg on following days 1, 8, 15, 22, 29, and 36, and ActD injection 475 mcg on following days 1, 15, and 29. Bilateral NSS was performed in a single operation to remove all macroscopic tumors.	Massive reduction (>50%) in the size of renal masses observed on CT scan.	Nerli et al., (2018) [7]
A five-year-and-10-month-old male had coexistence of WT and papillary RCC with a malignant tumor on the left kidney and clinical symptoms of hematuria, weight loss, fever, and an abdominal mass. Also, a CT scan revealed scattered calcifications on the left kidney. The NWTS classification of WT was stage I and RCC was grade 2 and T1aN0M0 (TNM staging).	RN of the left kidney was performed for stage I WT with complete resection of the tumor. Postoperation after one month, chemotherapy was given (vincristine + dactinomycin (EE-4A regimen))	At the 69-month follow-up, no recurrence or metastasis of the tumor was observed.	Zou et al., (2019) [8]

In many of the cases of Wilms’ tumor, preoperative chemotherapy leads to a significant decrease in tumor size, thus allowing subsequent renal salvage surgery (NSS) [7,9]. In our patient, unfortunately, the right-sided tumor reduction following chemotherapy was less than 50%, making NSS for the right kidney impossible. Hence, he underwent a right-sided radical nephrectomy with partial nephrectomy on the left side.

After 12 weeks, a long chemotherapy repeat CT scan revealed a marked interval reduction of the right kidney mass. However, the left kidney mass responded poorly to chemotherapy.

A literature review revealed that the coexistence of Wilms’ tumor with other renal cancer, although rare, is not unheard of [8]. In our case, the right-sided mass showed a marked interval reduction of right-sided kidney mass post-chemotherapy; however, the left-sided mass did not show such a favorable response after chemotherapy. Hence, we considered the presence of coexisting benign kidney disease in the left kidney as a cause of poor response to chemotherapy. However, this was ruled out by a nuclear scan, preoperative ultrasound-guided biopsy of the left renal mass, and frozen section examination. The result showed the presence of a round blue cell tumor. We proceeded with the surgical removal of the left kidney. Biopsy results confirmed the diagnosis of Wilms’ tumor.

BWT is considered an independent risk factor for renal failure [10]. Hence, the benefit of NSS in BWT patients is easily understandable as it facilitates the preservation of functional renal tissue and thus maintains adequate renal function [10].

## Conclusions

The rare pathological findings from the young patient with BWT showed varied responses to preoperative chemotherapy on either side of the kidneys, which followed another chemotherapy treatment even after performing NSS. Also, multiple pathologies of other kidney diseases such as a benign tumor or congenital condition along with BWT might affect the chemotherapy regimen. In our opinion, the differences observed throughout the treatment following preoperative chemotherapy in BWT patients should be explored thoroughly, keeping in mind the possibility of coexisting benign diseases or Wilms’ tumor with different immune histopathology, as found in our case.
